# A Remarkable Man

**Published:** 1892-05

**Authors:** 


### A Remarkable Man.

We enjoyed the privilege a short time ago of a brief visit with Dr. J. A. Robinson, of Jackson, Mich. (familiarly known as Uncle Jerry). We found him in excellent health, and with the spirits and buoyancy of a young man of twenty, though he is now within a few days of eighty years of age, and at work at the chair apparently as much interested in what he was doing as a beginner making his first fillings. Some fillings were shown, in the mouth of a patient, made by him within the last three years, which seemed as perfect and secure as any fillings could be.

He is probably the oldest practitioner in Michigan, having been in practice about fifty-four years. He studied dentistry in Boston, worked there for several years, afterward came to Cleveland, Ohio, where, in connection with his brother, he conducted a very successful practice for a number of years; then removed to the city of his present home, where he has for thirty years been in continuous practice and manifests an unusual interest in all that pertains to dentistry. A few years ago he devised a remedy, which bears his name, for the treatment of certain phases of diseased gums, which perhaps has no superior as a remedy for the affections to which he applies, and for which he recommends it; it is a heroic but very efficient remedy, as will be readily understood when its constituents are recognized, which are carbolic acid and caustic potash, properly combined and prepared.

Though Dr. Robinson has been and is greatly interested in all that pertains to his profession, he has always been interested in and given attention to many outside things. He is by no means a man of one idea. He has been nearly all his life actively engaged in Christian work, and that in various directions, in Church, in Sunday-school, and in all that pertains to public morals and well-being. He has been much interested in political matters, though he never held nor sought a political office. He has been a voluminous writer upon subjects of political and public interest; though his writings were by no means confined to politics, he has written on scientific subjects, moral, religious, social and professional questions. His productions have been published in newspapers, periodicals and the various journals of the day; these he has carefully collected and arranged, and they constitute a large volume. Though at the age of eighty, he is writing nearly as much and as well, perhaps better, than ever before. It is generally presumed that soon after sixty-five or seventy years of age men begin to lose interest, energy and ability in all the affairs of life; Dr. Robinson seems to be an exception to this idea. His life has been one of correct habits and freedom from many things that injure if not destroy, in so great a number of instances.

